# The Association Between Dry Eye and Sleep Quality Among the Adult Population of Saudi Arabia

**DOI:** 10.7759/cureus.22736

**Published:** 2022-03-01

**Authors:** Rahaf Almutairi, Sarah Algezlan, Rawan Bayamin, Shawg Alrumaih, Renad Almutairi, Rahaf Alkahtani, Abdulrahman A Almazrou

**Affiliations:** 1 Medical School, College of Medicine, Imam Mohammad Ibn Saud Islamic University, Riyadh, SAU; 2 Ophthalmology, Imam Mohammad Ibn Saud Islamic University, Riyadh, SAU

**Keywords:** work productivity, quality of sleep, ocular surface disease, meibomian gland dysfunction, dry eye

## Abstract

Background and objective

Dry eye disease (DED) is one of the most prevalent ocular diseases worldwide. DED symptoms can result from disturbances to the homeostasis of the middle tear film layer (aqueous layer), including inflammation, pain, and eye discomfort, which can have a negative impact on individuals’ quality of life and daily activities. Sleep disorders are highly prevalent among patients with DED, and the incidence of sleep disturbances in DED patients has been reported to be as high as 40%. Decreased sleep quality can aggravate dry eye symptoms by increasing tear osmolarity and decreasing tear production. In this study, we aimed to investigate the association between DED and sleep quality in the adult population of Saudi Arabia.

Methods

This cross-sectional study was conducted among adult patients aged 18 years and above in Saudi Arabia in August 2021. A validated Arabic version of the Pittsburgh Sleep Quality Index (PSQI) was used to evaluate sleep quality, and the Ocular Surface Disease Index (OSDI) questionnaire was employed to diagnose DED. Data collection and analysis were performed using the SPSS Statistics software (IBM, Armonk, NY).

Results

A total of 234 subjects were analyzed, and 59.8% of the participants were women. Our tool suggested that 71.4% of the included participants had severe DED, 15% had moderate DED, and 13.7% had mild DED. However, 40.6% of the participants reported that they had not been diagnosed with DED previously and 34.6% had no previous DED symptoms. The mean total PSQI score was 8.63 ±2.23, with the highest score recorded for component 2: sleep latency (1.73) and the lowest score recorded for component 4: habitual sleep efficiency (0.20). Poor sleep quality as assessed by PSQI showed a significant positive correlation with the severity of DED as assessed by OSDI.

Conclusion

The significant positive correlation between poor sleep quality and DED indicated that patients with DED had a higher risk of poor sleep quality compared to healthy patients. Patients with DED should be educated about the steps and techniques to improve their sleep patterns.

## Introduction

Dry eye disease (DED) is a multifactorial chronic disease of the ocular surface that is caused by abnormalities in the tear matrix. This condition presents with symptoms such as eye discomfort, pain, burning, foreign body sensation, inflammation, photosensitivity, visual disturbance, and, in rare cases, it may lead to loss of vision [1,2]. DED is considered one of the most prevalent ocular diseases worldwide, with the reported prevalence ranging from 4.4 to 50% [3-5]. The prevalence of DED has been reported to be 32.1% in Saudi Arabia [2]. DED is caused by disturbances in the functional unit of the eye consisting of the ocular surface, meibomian glands, the main lacrimal gland, and the innervation between them [6].

DED has been classified into two types: episodic and chronic; of the two, episodic DED results from multiple causes, such as prolonged visual tasks with reduced blinking, and it progresses to chronic DED when these symptoms persist [7]. This condition has been shown to have a negative impact on patients’ daily activities, such as watching television and reading, as well as work productivity [8]. More severe consequences of DED include difficulties in focusing and driving at night, which were reported in 47.5% and 64.7% of the population, respectively [7].

The term “sleep disorders” is used to refer to multiple diseases that affect sleep quality, such as obstructive sleep apnea and insomnia [9]. The prevalence of sleep disturbances has been increasing recently, with 30-60% of American adults and adolescents reporting insufficient amounts of sleep, respectively [10,11]. A survey of sleep disorders among workers in Saudi Arabia showed that among 116 respondents, 6.9% reported good sleep quality, while the majority (93.1%) reported experiencing sleep disorders, with insomnia being the most frequently reported sleep disorder (72%) [12]. Another recent investigation has suggested that the severity of DED is highly correlated with a poorer quality of life. Moreover, a previous study has shown that low sleep quality was present in more than 40% of patients with DED [13,14]. Reduction in tear secretion and increased tear osmolarity is one explanation for the link between DED and sleep deprivation [15]. The relationship between DED and sleep quality has been extensively studied in the literature, with many studies conducted in the United States, South Korea, Japan, and Turkey [16-18]. However, to the best of our knowledge, no such study has been conducted in Saudi Arabia so far. In light of this, this study aimed to investigate the association between DED and sleep quality in an adult population in Saudi Arabia.

## Materials and methods

In this cross-sectional study, the calculated study sample using the OpenEpi web tool, with a 95% confidence level and an estimated prevalence of 32.1%, was 337 participants [2]. Before the data collection, we obtained institutional review board (IRB) approval from the Imam Muhammad Ibn Saud Research Ethics Committee in Riyadh City with approval number 183-2021; in addition, informed consent was obtained from all the study participants. We used social media platforms to distribute our questionnaire in August 2021. Our inclusion criteria were as follows: individuals aged ≥18 years with Ocular Surface Disease Index (OSDI) and Pittsburgh Sleep Quality Index (PSQI) scores greater than or equal to 13 and 5.5, respectively. The survey consisted of three sections: demographics, OSDI score, and PSQI score. The first section entailed demographic information such as sex, nationality, marital status, smoking status (current and former smokers and non-smokers), educational level, occupation, number of hours spent on work each day, past medical history (glaucoma, cataract, and refractive eye diseases such as myopia and hyperopia), and medication use (isotretinoin, oral corticosteroids, antidepressants, and others if any). Patients were classified into four categories based on age: 18-30 years, 31-40 years, 41-50 years, and >50 years.

We used the validated Arabic version of the OSDI [7] to diagnose DED. The OSDI is a 12-item questionnaire that assesses the severity of dry eye according to a score that ranges between 0 and 100. A score between 13 and 22 indicated mild DED, those between 23 and 32 indicated moderate DED, and scores greater than 33 indicated severe DED. Additionally, we included questions regarding previous corrective eye surgery, family history of DED, and contact lens use.

A validated Arabic version of the PSQI [18] was used to evaluate sleep quality. The PSQI consists of 19 questions involving the following seven components: subjective sleep quality, sleep latency, sleep duration, habitual sleep efficiency, sleep disturbance, sleep medication usage, and daytime dysfunction. Each component score ranged from 0 to 3, and the sum of all component scores yielded a global PSQI score ranging from 0 to 21, with greater scores indicating poorer sleep quality.

The collected data was refined, entered into, and analyzed using the SPSS Statistics software version 26 (IBM, Armonk, NY). Descriptive statistics (frequencies, percentages, measures of central tendency, and dispersion, where appropriate) were calculated for each item in the survey and for all the demographic variables. Means and standard deviations (±SD) were used to present continuous variables and proportions of discrete variables. The Pearson’s correlation test was used to determine the relationship between DED and sleep quality. A p-value ≤0.05 was considered statistically significant.

## Results

Out of the total 516 responses to our survey, only 234 met the inclusion criteria and were included in the analysis. Of the 234 participants, 59.8% were female, 43.2% were aged 18-30 years, and 46.2% were from the central region; 14.1% of the participants reported being current smokers, 7.3% were former smokers, 50.9% were married, and 44.9% were single. In terms of educational level, 66.7% of the participants had a university degree as the highest educational qualification while 34.6% were still students at the time of the study. As for the duration of work or education, 49.6% of the participants reported spending more than six hours daily on work or education, and 32.1% spent four to six hours daily (Table 1).

**Table 1:** Demographic characteristics of the participants (n=234)

Variables		N	%
Age (years)	18-30	101	43.2%
	31-40	46	19.7%
	41-50	42	17.9%
	Older than 50	45	19.2%
Sex	Male	94	40.2%
	Female	140	59.8%
Residency	Central	108	46.2%
	Western	66	28.2%
	Eastern	24	10.3%
	Northern	7	3.0%
	Southern	29	12.4%
Smoking	Current smoker	33	14.1%
	Former smoker	17	7.3%
	Non-smoker	184	78.6%
Marital status	Single	105	44.9%
	Married	119	50.9%
	Divorced	10	4.3%
	Widowed	0	0.0%
Educational level	Pre-secondary school	46	19.7%
	Secondary school	0	0.0%
	University	156	66.7%
	Higher education	32	13.7%
Profession	Student	81	34.6%
	Office work	58	24.8%
	Outdoor work	27	11.5%
	Retired	30	12.8%
	Other	38	16.2%
Working hours	Less than 4 hours daily	43	18.4%
	4-6 hours	75	32.1%
	More than 6 hours daily	116	49.6%

**Figure 1:**
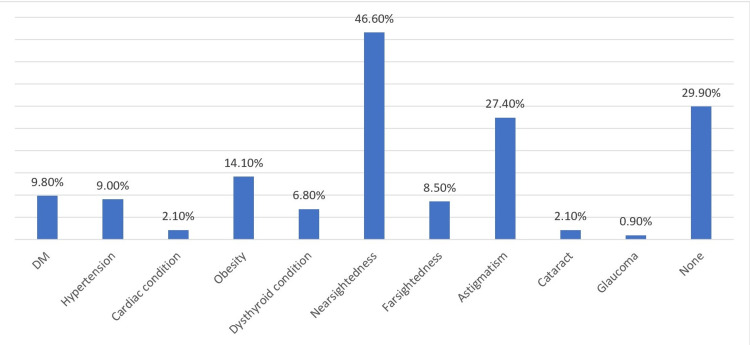
Frequency of medical conditions DM: diabetes mellitus

**Figure 2:**
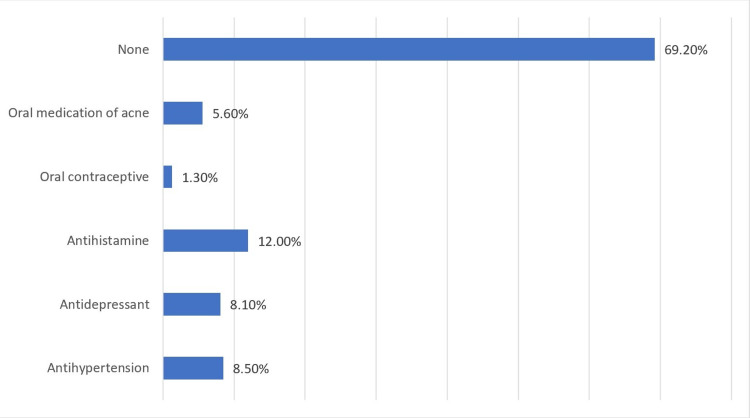
The prevalence of medication usage

As shown in Figure 1, 29.9% of the participants reported having no other medical conditions; however, 46.6% reported having nearsightedness and 27.4% reported astigmatism. The prevalences of diabetes mellitus (DM), hypertension, and obesity were 9.8%, 9%, and 14.1%, respectively. Moreover, while 69.2% of the participants reported not taking any medications, 12% reported using antihistamines, 8.5% used antihypertensive drugs, 8.1% used antidepressants, and 5.6% used oral medications for acne (isotretinoin) (Figure 2).

Our tool suggested that 71.4% of the participants had severe DED, 15% had moderate DED, and 13.7% had mild DED. However, 40.6% of the participants were not diagnosed priorly with DED, and 34.6% reported having no previous DED symptoms. Nevertheless, 61.5% of the participants reported using eye lubricant drops, with 19.2% reporting daily use and 21.8% reporting weekly use. Moreover, 37.2% of the participants had a family history of DED, 15.8% had undergone vision correction, and 22.2% reported using contact lenses (Table 2).

**Table 2:** The prevalence of DED among the participants DED: dry eye disease; OSDI: Ocular Surface Disease Index

Variables		N	%
OSDI	Mild	32	13.7%
	Moderate	35	15.0%
	Severe	167	71.4%
Have you been diagnosed with DED?	Yes	139	59.4%
	No	95	40.6%
When were you diagnosed with DED?	During the last 6 months	40	17.1%
	During the past year	28	12.0%
	1-5 years	51	21.8%
	More than 5 years	34	14.5%
	I have not been diagnosed	81	34.6%
Do you use an eye lubricant?	Yes	144	61.5%
	No	90	38.5%
Rate of eye lubricant usage	Once weekly	51	21.8%
	2-4 times weekly	33	14.1%
	5-6 times weekly	12	5.1%
	Daily	45	19.2%
	Not using	93	39.7%
Family history of DED	Yes	87	37.2%
	No	147	62.8%
Have you undergone vision correction?	Yes	37	15.8%
	No	197	84.2%
Do you use contact lenses?	Yes	52	22.2%
	No	182	77.8%

Table 3 shows the mean PSQI score as well as its component scores, with higher scores indicating poorer sleep quality. The mean total PSQI score was 8.63 ±2.23; the highest score was recorded for component 2: sleep latency (1.73), and the lowest score was recorded for component 4: habitual sleep efficiency (0.20) (Table 3).

**Table 3:** Mean and standard deviation of participants’ PSQI and PSQI component scores PSQI: Pittsburgh Sleep Quality Index

	Mean	Standard deviation
Component 1: subjective sleep quality	1.30	0.89
Component 2: sleep latency	1.73	0.87
Component 3: sleep duration	1.52	0.97
Component 4: habitual sleep efficiency	0.20	0.68
Component 5: step disturbance	1.55	0.62
Component 6: use of sleep medication	0.39	0.85
Component 7: daytime dysfunction	1.24	0.77
Total PSQI	8.63	2.23

Table 4 illustrates the possible demographic factors that could have affected the severity of DED and sleep quality. Although no demographic factors seemed to have a significant influence on the severity of DED or sleep quality, the severity of DED appeared to be slightly higher in younger participants. Moreover, severe DED and poorer sleep quality were slightly more prevalent among females than males (72.9% of females had severe DED with a PSQI score of 8.86 vs. 69.1% of males with a PSQI score of 8.3).

**Table 4:** The association of demographic factors with prevalence of dry eye and sleep quality OSDI: Ocular Surface Disease Index; PSQI: Pittsburgh Sleep Quality Index

		OSDI							PSQI	
		Mild		Moderate		Severe		P-value	Mean	P-value
		N	%	N	%	N	%			
Age (years)	18-30	9	8.9%	17	16.8%	75	74.3%	0.247	8.44	0.395
	31-40	7	15.2%	6	13.0%	33	71.7%		8.61	
	41-50	5	11.9%	8	19.0%	29	69.0%		9.14	
	Older than 50	11	24.4%	4	8.9%	30	66.7%		8.62	
Sex	Male	16	17.0%	13	13.8%	65	69.1%	0.466	8.30	0.06
	Female	16	11.4%	22	15.7%	102	72.9%		8.86	
Smoking	Current smoker	5	15.2%	5	15.2%	23	69.7%	0.970	8.52	0.944
	Former smoker	3	17.6%	3	17.6%	11	64.7%		8.71	
	Non-smoker	24	13.0%	27	14.7%	133	72.3%		8.65	
Martial status	Single	9	8.6%	14	13.3%	82	78.1%	0.185	8.57	0.141
	Married	22	18.5%	20	16.8%	77	64.7%		8.57	
	Divorced	1	10.0%	1	10.0%	8	80.0%		10.00	
Profession	Student	7	8.6%	15	18.5%	59	72.8%	0.698	8.46	0.737
	Office work	7	12.1%	9	15.5%	42	72.4%		8.52	
	Outdoor work	5	18.5%	3	11.1%	19	70.4%		8.70	
	Retired	5	16.7%	3	10.0%	22	73.3%		9.07	
	Other	8	21.1%	5	13.2%	25	65.8%		8.79	
Working hours	Less than 4 hours daily	6	14.0%	5	11.6%	32	74.4%	0.828	8.79	0.697
	4-6 hours	8	10.7%	13	17.3%	54	72.0%		8.73	
	More than 6 hours daily	18	15.5%	17	14.7%	81	69.8%		8.51	
Comorbidities	Yes	18	11.0%	28	17.1%	118	72.0%	0.101	8.76	0.195
	No	14	20.0%	7	10.0%	49	70.0%		8.34	

Table 5 illustrates the significant positive correlation between sleep quality as assessed by the PSQI and the severity of DED as assessed by the OSDI. Poor sleep quality indicated that patients would have more severe DED, and patients with severe DED would be expected to have worse sleep quality.

**Table 5:** Correlation between OSDI and PSQI scores *Significant correlation (2-tailed) OSDI: Ocular Surface Disease Index; PSQI: Pittsburgh Sleep Quality Index

		OSDI	PSQI
OSDI	Pearson correlation	1	.255^*^
	Significance (2-tailed)		0.000
	N	234	234
PSQI	Pearson correlation	.255^*^	1
	Significance (2-tailed)	0.000	
	N	234	234

## Discussion

This study aimed to assess the influence of DED on sleep quality by using validated tools to analyze each variable. The findings revealed a significant positive correlation between poor sleep quality and the severity of DED. Thus, patients with more severe DED would experience poorer-quality sleep patterns.

In the study by Magno et al., the authors found that across all demographic data, sleep quality was significantly poorer in patients with dry eyes, and that dry eyes were still associated with decreased sleep quality after treatment for other related diseases [1]. Ayaki et al. found that dry eye treatment significantly improved sleep quality in newly diagnosed DED patients, confirming the influence of DED on sleep quality, which is consistent with our findings [19]. Furthermore, our study showed that this link is two-dimensional, indicating that poor sleep quality can also lead to dry eye conditions and increase their severity. A previous study in mice has found that sleep deprivation increased corneal epithelial cell defects, decreased aqueous tear secretion, and induced squamous metaplasia of the corneal epithelium [20]. In people-based studies in Singapore (n=3,303), Lim et al. found that daytime sleepiness, clinical insomnia, and short sleep periods (<5 hours) were associated with dry eyes, after correcting for socioeconomic and medical factors [21]. In a systematic review conducted by Au et al., the authors found that among 16,370 participants, DED patients had higher scores than control participants for both the PSQI and the Epworth Sleepiness Scale and they showed shorter duration of sleep, more sleep disturbances, and a higher prevalence and severity of sleep problems [22].

Previous studies have reported that sleep disturbances caused by obstructive sleep apnoea syndrome, which is characterized by recurrent complete or partial upper airway obstruction during sleep, are linked to DED [23-25]. DED is thought to be associated with the increased surface tension forces of the salivary and upper airway mucous, which participate in the pathophysiology of obstructive sleep apnea [26-28]. However, no significant associations were found between measures of sleep disruption, the severity of sleep apnea, and subjective sleep quality [29]. These results indicate the importance of examining other potential explanations, such as psychological factors, because multiple studies have shown inconsistencies between subjective and objective evaluations of sleep [29,30]. Positive and negative affective states are associated with physical health consequences, and there is considerable evidence regarding the association of negative affective states with both sleep duration and quality [31-33]. Thus, for example, depression, which is one of the most common mental health conditions, is known to be associated with sleep disturbances [34], which is in line with our study, in which 8.1% of the participants reported using antidepressant medication. Moreover, in this study, 61.5% of the patients reported using eye lubricant drops to reduce DED symptoms. Tang et al. conducted an animal trial to provide an explanation for the impact of sleep deprivation on the severity of DED and showed that sleep deprivation induces dry eye through abnormal superficial corneal epithelial cell (SCEC) microvilli morphology, which is caused by sequential downregulation of PPARα, TRPV6 expression, and ezrin phosphorylation status in mice [35].

In a study about the primary medications used for DED symptoms, the authors found that ocular lubricants were the main pharmacotherapeutic agents used by 65.9% of the patients with DED [36]. This study did not reveal any significant correlations between any of the demographic factors and the severity of DED or sleep quality. This could indicate a reduced importance of demographic factors and confirm a significant positive correlation between the severity of DED and poor sleep quality. In another study, the authors found that females had significantly higher severity of DED as well as lower sleep hours and anxiety, which is in agreement with our study; they also reported that age had no impact on DED [37]. Many previous studies have reported that DED severity increased with age [38-42], while others did not find any correlation [43-46].

This study has some unavoidable limitations. Despite using validated questionnaires to diagnose DED and sleep disturbance, the findings may have been influenced by some personal biases, wherein participants may not have answered all questions honestly. Moreover, our inclusion criteria resulted in a small study sample despite an extended data capture time.

## Conclusions

Based on our findings, poor sleep quality had a significant positive correlation with the severity of DED. Therefore, patients with DED have a higher risk of poor sleep quality than normal patients. Patients with DED should be educated on the steps and techniques to improve their sleep patterns.
